# Insulin resistance has closer correlation with the occurrence of metabolic dysfunction associated steatotic liver disease diagnosed by liver biopsy

**DOI:** 10.3389/fmed.2024.1384927

**Published:** 2024-11-15

**Authors:** Weihua Cao, Tingting Jiang, Wen Deng, Shiyu Wang, Xinxin Li, Ziyu Zhang, Lu Zhang, Yao Lu, Min Chang, Ruyu Liu, Shuling Wu, Ge Shen, Yuanjiao Gao, Hongxiao Hao, Xiaoxue Chen, Leiping Hu, Mengjiao Xu, Wei Yi, Yao Xie, Minghui Li

**Affiliations:** ^1^Department of Hepatology Division 2, Beijing Ditan Hospital, Capital Medical University, Beijing, China; ^2^Department of Gynecology and Obstetrics, Beijing Ditan Hospital, Capital Medical University, Beijing, China; ^3^Department of Hepatology Division 2, Peking University Ditan Teaching Hospital, Beijing, China

**Keywords:** metabolic dysfunction associated steatotic liver disease, hyperuricemia, metabolic syndrome, liver biopsy, low-density lipoprotein, hyperinsulinemia

## Abstract

**Objective:**

To explore any correlation between serum urate (SU) level or insulin resistance (IR) and metabolic dysfunction associated steatotic liver disease (MASLD) in patients with metabolic syndrome (MS).

**Methods:**

Data from all MASLD patients, diagnosed by liver biopsy, were enrolled and divided into MASLD alone group and MASLD with MS group. They were subdivided into hyperuricemia group and normal SU group to find correlation between SU/IR and MASLD in patients with MS and independent risk factors for MASLD.

**Results:**

Data from 539 MASLD patients were analyzed. Body mass index (BMI) (*p* = 0.000), waist circumference (WC) (*p* = 0.004), and low-density lipoprotein (LDL) (*p* = 0.000) were dramatically higher in MASLD with MS group than those with MASLD alone; MASLD with MS patients had significantly more family history of diabetes (*p* = 0.000) and hypertension (*p* = 0.000) than patients with MASLD alone. Height (*p* = 0.000), weight (*p* = 0.000), BMI (*p* = 0.000) and WC (*p* = 0.001), and LDL (*p* = 0.007) were dramatically higher in hyperuricemia patients than those with normal SU. SU was inversely associated with age (*p* = 0.000) and high-density lipoprotein (HDL) (*p* = 0.003), and positively correlated with weight (*p* = 0.000), BMI (*p* = 0.000) and WC (*p* = 0.000), TG (*p* = 0.000), and LDL (*p* = 0.000). Logistic Regression analysis showed that age (*p* = 0.031), TG (*p* = 0.002), LDL (*p* = 0.010), HbA1c (*p* = 0.026), and family history of hypertension (*p* = 0.000) may be independent risk factors for MASLD in patient with MS.

**Conclusion:**

Insulin resistance (IR) in MASLD patients with MS, but not higher SU levels, has closer correlation with the occurrence of MASLD in patients with family history of hypertension and diabetes having higher BMI, LDL, HbA1c.

## Introduction

The metabolic dysfunction associated steatotic liver disease (MASLD), a major health burden, the most common cause of chronic liver disease all over the world, has lately risen with the estimated worldwide prevalence of about 38% (1) of the general population (2–4). MASLD is a disease caused by the interaction of multiple factors such as genetics, diet, and lifestyle (5). It is characterized by excessive accumulation of triglyceride within the cytoplasm of hepatocytes (exceeding 5% of liver weight) due to both increased inflow of free fatty acids and *de novo* hepatic lipogenesis and insufficient mitochondrial capacity for beta oxidation in individuals without alcohol consumption (6). Its clinical manifestations include metabolic dysfunction associated hepatic steatosis (MAHS; simple benign condition of MAFL), metabolic dysfunction associated steatohepatitis (MASH; inflammatory subtype of MAFL with lobular inflammation and apoptosis), that can progress to liver cirrhosis, liver failure and liver cancer if left untreated (7, 8). Patients with MASLD often have systemic hypertension, dyslipidaemia, insulin resistance/diabetes which are prognostic variables common to the metabolic syndrome (MS) (9). MASLD is the hepatic manifestation of the MS which is often associated with abnormal liver enzyme levels such as elevated levels of alanine aminotransferase (ALT) and aspartate aminotransferase (AST) (10, 11). Though growing evidence suggest bidirectional relationship between MASLD and MS because MASLD can predispose to MS, but hepatic steatosis reportedly can occur independently of insulin resistance (12–15). MASLD can occur in individuals who are not obese (16, 17). In studies of hepatic steatosis, mice over-expressing DGAT2, an enzyme that catalyzes the final step of hepatic triglyceride biosynthesis, was demonstrated to develop hepatic steatosis with normal plasma glucose and insulin levels and normal insulin tolerance (12). The primary therapy for most patients with MASLD is weight loss. However, pioglitazone (an anti-diabetic medication) (18, 19), and Glucagon-like peptide-1 (GLP-1) receptor agonists (20, 21) have shown promising hepatic outcomes in patients with MASLD. For MASLD diagnosis, liver biopsy is the gold standard to diagnose any form of hepatic inflammation, fibrosis or injury associated with it. Nevertheless, owing to its invasive nature, it has not been routinely used to diagnose various liver diseases.

MASLD was demonstrated as a manifestation of metabolic syndrome (MS) (22, 23) which has high incidence rate in the adult population leading to the increase in the cost of the global public health. MS is a non-communicable complex pathophysiologic state of a group of interrelated diseases characterized by at least any three or more of the following conditions: abdominal obesity (greater waist circumference), high fasting blood glucose, insulin resistance, high triglycerides, lower high-density lipoprotein cholesterol (HDL), higher low-density lipoprotein cholesterol (LDL), high blood pressure and hypertension (24). MS has become the major health hazard of modern world caused primarily by increased consumption of high calorie and low-fiber fast food, sedentary lifestyle of reduced physical activity including genetic/epigenetic makeup of individual that can lead to cardiovascular and cerebrovascular diseases (25). The liver plays a central role in metabolic syndrome due to its role in glucose and triglyceride overproduction because two key components of MS, glucose and triglycerides, are overproduced by the fatty liver.

Serum urate (SU) is the end-product of purine (endogenous and exogenous) metabolism in humans and the great apes because of loss-of- function mutations during primate evolution in the gene of uricase enzyme, that oxidizes uric acid to more soluble allantoin (26, 27). Due to uricase inactivation the SU level is 7 to 8-fold higher in humans (≈240–360 μM) compared to other mammals (≈30–50 μM in mice) (27). Thus, higher SU levels may have selective advantage in the evolution of hominids, may be related to memory with less SU level linked to neurodegenerative disorders, including Alzheimer’s Disease (28). Approximately two-third of the SU is produced endogenously, and the remaining comes from dietary purines (29). The normal SU level in humans is the result of net balance between biosynthesis of SU primarily in liver, reabsorption of urate in renal proximal tubules and secretion in the renal tubule and intestine (30). Most SU is filtered freely in the kidneys, with roughly 90% of the urate from glomerular filtrate is reabsorbed via urate transporters in the proximal tubule (31). About 70% of the total uric acid from our body passes through the kidneys and the rest via intestinal and biliary secretion (29). Ultimately, after urate reabsorption, only 3–10% of the filtered urate is excreted in the urine (32). Abnormalities in SU metabolism and its decreased excretion by the kidneys are one of the major causes of hyperuricemia and gout development (33). Dysregulation of xanthine oxidoreductase (XOD), the enzyme that catalyzes the endogenous production of SU primarily in liver and urate transporters (34) that reabsorb urate in renal proximal tubule and secrete urate in renal tubule and intestine, and their genetic variabilities are the major causes for the development of hyperuricemia. SU is elevated in metabolic syndrome (MS) and diabetes (35) as a consequence of insulin resistance and the effects of insulin to reabsorb more urate resulting in reduced urinary urate excretion (36). It is debatable that elevated SU levels can lead to insulin resistance. In fact, there is a positive relationship between serum insulin and elevated SU levels, in healthy volunteers and people with diabetes (36). Insulin resistance also leads to impaired SU excretion at a low urinary pH, contributing to the formation of urate stones (37). These genetic data are consistent with a causal role of insulin to control SU levels (36). Insulin resistance is considered the major mechanism in the development and progression of MASLD/MASH as a result of impaired insulin signaling that leads to increase intracellular fatty acid-derived metabolites such as diacylglycerol, fatty acyl CoA or ceramides (6). It is debatable whether elevated level of SU is the causative factor of MASLD, because allopurinol (an inhibitor of XOD) treatment to reduce serum urate level was shown to significantly increase the triglyceride values (38). However, febuxostat (another inhibitor of XOD) treatment, was shown to suppress the development of nonalcoholic steatohepatitis in a rodent model (39).

Previous studies on MASLD, MS, and blood uric acid levels have rarely been based on liver biopsy for diagnosis of MASLD patients. In this study, MASLD patients were diagnosed using liver biopsy to find any correlation between MASLD and insulin resistance or SU level using prognostic variables from MASLD patients without or with MS.

## Materials and methods

### Subjects

Patients admitted to Beijing Ditan Hospital from October 2008 to December 2018 underwent liver biopsy, for the diagnosis of MASLD. Inclusion criteria: MASLD diagnosis of all patients by liver biopsy. Exclusion criteria: (1) Liver diseases caused by alcoholic hepatitis, autoimmune hepatitis etc.; (2) liver diseases caused by viral infections such as EB virus (Epstein–barr Virus, EBV), CMV (Cytomegalovirus), HIV (Human Immunodeficiency Virus); (3) mental diseases; (4) liver tumors. This study was approved by the Ethics Committee of Beijing Ditan Hospital and the ethics ID was Jing Di Lun Ke Zi 2018 No. 052-01.

### Diagnostic criteria for liver tissue biopsy and histopathology

A 16G liver puncture needle was used under ultrasound guidance for liver tissue puncture, and the length of the tissue specimen was required to be at least 1.0 cm (1.5–2.5 cm). Liver biopsy specimens were consecutively sliced and subjected to routine H–E, reticular fibrosis, and/or Masson staining. The Scheuer scoring system was used to evaluate the staging of liver fibrosis (S0–S4) and inflammation grading (G0–G4), with S3–S4 defined as advanced liver fibrosis. According to the Brunt grading system, fat degeneration was evaluated and divided into four levels: F0 (<5%), F1 (5–33%), F2 (33–66%), and F3 (≥66%). All pathological sections were independently observed and evaluated by two experienced pathologists. In case of any disagreement, a third pathologist was there for arbitration.

### Clinical index detection

Liver function (Wako Pure Chemical Industries, Ltd., JAPAN) and kidney function (Sekisui Medical CALCo, Ltd., JAPAN) were detected using Hitachi fully automated biochemical analyzer (Hitachi Ltd). International standardized ratio (Beckman coulter, America) was measured. In this study, the upper bound of alanine transaminase (ALT) Aspartate transaminase (AST) detection value is 40 U/L, the upper normal value of total bilirubin (TBIL) is 18.8 μmol/L, the upper bound of the Gamma-glutamyl transferase (GGT) detection value is 60 U/L, the upper limit of the Alkaline phosphatase (ALP) detection value is 125 U/L, and the lower bound of the normal albumin detection value is 40 g/L.

### Statistical analyses

All data were subjected to statistical analyses using Statistical Package for the Social Sciences (SPSS 26.0 software; Chicago, IL, USA), GraphPad Prism 6 and WPS Office version 5.5.1 (7991) software. Before performing the analysis, the Kolmogorov–Smirnov method was used to analyze all data for the normality test. The count data were shown using a descriptive analysis and a percentage, and the comparison of data between two groups was performed by the Fisher’s exact test or chi-square test. The statistical description of normally distributed data were expressed by the mean ± standard deviation (Mean ± SD), and the comparison of data between two groups was performed by two independent samples t-test; non-normally distributed data were described using the median (Q1, Q3), and comparison between groups were performed by the nonparametric M-U test. Univariate and multivariate Logistic regression were used to analyze risk factors for MASLD with MS. All statistical tests were used two-sided, statistically significant if *p* < 0.05.

## Results

### Basic clinical characteristics of patients with MASLD

In this retrospective study, we collected data of MASLD patients from the outpatient department of Beijing Ditan Hospital, Capital Medical University. Table 1 showed a total of 539 MASLD patients (325 males and 214 females, aged 39.56 ± 13.11 years) diagnosed by liver biopsy. Patients were grouped according to whether they had MS. Patients with MASLD alone and patients with MASLD combined with MS were 53.06% (286) and 46.94% (253), respectively. MASLD patients with MS were significantly older than patients with MASLD alone (*p* < 0.001), as is shown in Table 1. Height and weight were not significantly different between two groups, but BMI (*p* < 0.001) and waist circumference (*p* = 0.004) were greater in MASLD with MS than those with MASLD alone. There were no significant differences in ALT (Alanine Transaminase), TBIL (Total Bilirubin), and DBIL (Direct/conjugated Bilirubin) levels between two groups, but the AST (aspartate aminotransferase) levels in MASLD patients with MS (*p* = 0.031) were higher (73.30 ± 87.65 vs. 59.81 ± 55.28) than those in patients with MASLD alone. There were no significant differences in blood glucose, SU, and creatinine levels, and triglyceride and HDL between two groups, while the levels of total cholesterol (*p* < 0.001) and LDL (*p* < 0.001) were higher (cholesterol: 5.08 ± 1.11 vs. 4.71 ± 0.99; LDL: 3.09 ± 1.18 vs. 2.71 ± 0.77) in MASLD patients with MS than patients with MASLD alone. Hepatic stiffness (*p* = 0.007) was greater in patients with MASLD combined with MS (10.31 ± 7.77 vs. 8.72 ± 5.51) than that in patients with MASLD alone. Glycated hemoglobin (HbA1c) (5.96 ± 1.26 vs. 5.41 ± 1.04, *p* = 0.000) and glycated albumin (14.32 ± 3.93 vs. 12.83 ± 3.18, *p* < 0.001) were higher in MASLD patients with MS than those in patients with MASLD alone. However, there were no significant differences in insulin and C peptide levels between two groups which could be due to their low half lives in serum (approximately 30 min for C-peptide and 5–6 min for insulin). More MASLD patients with MS had a family history of diabetes (*p* < 0.001) and hypertension (28.85% vs. 11.89%, *p* < 0.001) than patients with MASLD alone. Economic status of MASLD patients with MS (36.36% vs. 50.35%, *p* < 0.001) was worse than that patients with MASLD alone. The percentage of patients taking lipid-lowering drugs in MASLD with MS group was significantly higher (5.14% vs. 1.40%) than the patients in NAFLD alone group (*p* = 0.013) (Table 1).

**Table 1:** Comparison of clinical characteristics of patients with MASLD alone and MASLD with MS.

Item	All patients *n* = 539	MASLD alone group *n* = 286	MASLD with MS group *n* = 253	*t* or *χ*^2^	*p* value
Male (*n*, %)	325 (60.30%)	187 (65.38%)	138 (54.55%)	6.588	0.010
Age (yrs) (mean ± SD)	39.56 ± 13.11	37.03 ± 12.57	42.42 ± 13.13	4.866	0.000
Height (m) (mean ± SD)	1.69 ± 0.08	1.69 ± 0.08	1.68 ± 0.08	−1.814	0.070
Weight (kg) (mean ± SD)	79.42 ± 10.79	78.71 ± 11.29	80.22 ± 10.15	1.627	0.104
BMI (mean ± SD)	27.89 ± 2.70	27.44 ± 2.87	28.41 ± 2.40	4.224	0.000
WC (m) (mean ± SD)	1.19 ± 0.13	1.18 ± 0.14	1.21 ± 0.11	2.921	0.004
ALT (U/L) (mean ± SD)	121.66 ± 15.24	120.54 ± 14.62	122.92 ± 15.93	0.146	0.884
AST (U/L) (mean ± SD)	66.14 ± 7.26	59.81 ± 5.52	73.30 ± 8.77	2.120	0.034
TBIL (μmol/L) (mean ± SD)	15.33 ± 9.0.39	15.60 ± 9.15	15.02 ± 9.66	−0.188	0.188
DBIL (μmol/L) (mean ± SD)	5.91 ± 8.83	5.99 ± 8.07	5.83 ± 9.63	−0.208	0.835
ALB (g/L) (mean ± SD)	45.71 ± 4.26	45.65 ± 4.26	45.77 ± 4.27	0.317	0.751
GGT (U/L) (mean ± SD)	113.73 ± 17.16	109.73 ± 14.78	118.25 ± 19.53	0.752	0.452
ALP (U/L) (mean ± SD)	101.29 ± 11.36	103.22 ± 13.43	99.11 ± 8.45	−0.228	0.820
Creatinine (μmol/L) (mean ± SD)	64.13 ± 15.23	64.89 ± 15.24	63.29 ± 15.20	−1.215	0.225
SU (μmol/L) (mean ± SD)	373.16 ± 11.21	374.10 ± 10.83	372.10 ± 11.65	−0.272	0.786
GLU (mmol/L) (mean ± SD)	8.62 ± 3.78	7.77 ± 3.51	9.57 ± 4.07	0.550	0.583
TC (mmol/L) (mean ± SD)	4.88 ± 1.07	4.71 ± 0.99	5.08 ± 1.11	3.997	0.000
TG (mmol/L) (mean ± SD)	2.14 ± 1.69	1.96 ± 1.62	2.34 ± 1.74	−2.670	0.008
HDL (mmol/L) (mean ± SD)	1.10 ± 0.79	1.10 ± 0.33	1.11 ± 1.11	−0.127	0.899
LDL (mmol/L) (mean ± SD)	2.88 ± 1.00	2.71 ± 0.77	3.09 ± 1.18	4.415	0.000
PTA (%) (mean ± SD)	99.72 ± 14.42	100.10 ± 12.91	99.29 ± 15.96	−0.645	0.519
INR (mean ± SD)	1.35 ± 5.08	1.14 ± 2.42	1.58 ± 6.95	1.026	0.305
Hepatic stiffness (mean ± SD)	9.47 ± 6.71	8.72 ± 5.51	10.31 ± 7.77	2.712	0.007
HbA1c (%) (mean ± SD)	5.67 ± 1.18	5.41 ± 1.04	5.96 ± 1.26	5.334	0.000
GA (mmol/L) (mean ± SD)	13.54 ± 3.63	12.83 ± 3.18	14.32 ± 3.93	4.639	0.000
Insulin (μU/mL) (mean ± SD)	17.18 ± 0.5.98	18.80 ± 8.14	15.40 ± 4.91	−0.658	0.511
C-peptide (ng/mL) (mean ± SD)	4.39 ± 2.88	4.23 ± 2.70	4.56 ± 3.06	1.274	0.203
Smoking	98 (18.18%)	48 (16.78%)	50 (19.76%)	0.801	0.371
Family history of diabetes	108 (20.04%)	33 (11.54%)	75 (29.64%)	27.467	0.000
Family history of hypertension	107 (19.85%)	34 (11.89%)	73 (28.85%)	24.286	0.000
Good economic status	236 (43.78%)	144 (50.35%)	92 (36.36%)	10.669	0.001
Sedentary lifestyle	512 (94.99%)	269 (94.06%)	243 (96.05%)	1.119	0.290
High caloric diet	415 (76.99%)	212 (74.13%)	203 (80.24%)	2.831	0.092
Taking lipid-lowering drugs	17 (3.15%)	4 (1.40%)	13 (5.14%)	6.147	0.013

ALT, alanine aminotransferase; AST, aspartate aminotransferase; Tbil, total bilirubin; ALB, albumin; ALP, alkaline phosphatase; GGT, glutamyl transferase; SU, serum urate; GLU, glucose; TC, total cholesterol; TG, triglycerides; HDL, high-density lipoprotein; LDL, low-density lipoprotein; HbA1c, Glycated hemoglobin; GA, Glycated albumin; INR, International normalized ratio; PTA, prothrombin activity.

### Comparison of MS components between hyperuricemia and normal SU patients

In this study, data from 539 MASLD patients, confirmed by liver biopsy, were collected, in which blood urate data from two patients were missing. Therefore, data from 537 patients were included for the comparison of MS components between hyperuricemia subgroup and the normal SU subgroup. In this group of MASLD patients, the proportion of male patients was higher (60.34% male and 39.66% female) than that of female patients, and there was significant statistical difference in the proportion of male patients between two groups (hyperuricemia vs. normal SU group) (*p* = 0.001). Average age of hyperuricemia patients was significantly lower than those with normal serum urate level, as is shown in Table 2. Height (*p* = 0.000), weight (*p* < 0.001), BMI (*p* < 0.001) and waist circumference (*p* = 0.001) were significantly higher in hyperuricemia patients than those with normal serum urate. There was no significant difference in transaminase level between two groups. However, the albumin (ALB) level (*p* < 0.001), glycated albumin level (a marker of glycemic control) (*p* = 0.044) and creatinine level (*p* < 0.001) in hyperuricemia patients were significantly higher than that of normal SU patients. Although there was no significant difference in triglyceride (TG) level between two groups, total cholesterol (TC) (*p* = 0.044) and low-density lipoprotein (LDL) levels (*p* = 0.007) were significantly higher in hyperuricemia group than those in normal blood urate group. Interestingly, in Table 2 of our study, MASLD patients with hyperuricemia had lower HDL level (*p* = 0.045) with higher platelet to HDL ratio (PHR) (*p* = 0.033), than that of MASLD patients with normal serum urate level. The glycated albumin (GA) of hyperuricemia patients was markedly lower than that of normal blood urate patients (*p* = 0.044); the percentage of hyperuricemia patients with family history of hypertension (*p* = 0.001) and high-calorie diet (*p* < 0.001) was markedly lower than that of normal blood urate patients in Table 2. In Table 2 of this study, MASLD patients with hyperuricemia have lower HDL level (1.04 ± 0.24 mmol/L) with the chance of higher PHR, than that of MASLD patients with normal serum urate level.

**Table 2:** Comparison of MS components between MASLD patients with hyperuricemia and MASLD patients with normal SU levels.

Item	Hyperuricemia group *n* = 207	Normal SU group *n* = 330	*t* or *χ*^2^	*p* value
Male (*n*, %)	143 (69.08%)	181 (54.85%)	10.769	0.001
Age (yrs) (mean ± SD)	34.93 ± 12.47	42.41 ± 12.68	6.695	0.000
Height (m) (mean ± SD)	1.70 ± 0.08	1.68 ± 0.08	−3.284	0.001
Weight (kg) (mean ± SD)	82.81 ± 10.59	77.32 ± 10.40	−5.909	0.000
BMI (mean ± SD)	28.60 ± 2.54	27.46 ± 2.72	−4.853	0.000
WC (m) (mean ± SD)	1.24 ± 0.12	1.17 ± 0.13	−6.066	0.000
ALT (U/L) (mean ± SD)	123.04 ± 11.65	120.98 ± 17.16	−0.165	0.869
AST (U/L) (mean ± SD)	67.84 ± 6.47	65.15 ± 7.74	−0.434	0.664
TBIL (μmol/L) (mean ± SD)	14.99 ± 9.22	15.56 ± 12.60	0.570	0.569
DBIL (μmol/L) (mean ± SD)	5.58 ± 7.19	6.13 ± 9.75	0.707	0.480
ALB (g/L) (mean ± SD)	46.85 ± 3.71	44.98 ± 4.44	−5.048	0.000
GGT (U/L) (mean ± SD)	108.72 ± 17.92	117.24 ± 16.73	0.549	0.583
ALP (U/L) (mean ± SD)	90.20 ± 5.31	108.46 ± 13.86	1.813	0.069
Creatinine (μmol/L) (mean ± SD)	68.57 ± 15.17	61.35 ± 14.61	−5.490	0.000
SU (μmol/L) (mean ± SD)	480.42 ± 7.97	305.88 ± 6.88	−26.012	0.000
GLU (mmol/L) (mean ± SD)	5.97 ± 1.23	6.51 ± 2.15	−3.313	0.001
TC (mmol/L) (mean ± SD)	5.00 ± 1.01	4.81 ± 1.10	−2.014	0.044
TG (mmol/L) (mean ± SD)	2.30 ± 1.67	2.12 ± 2.17	1.007	0.314
HDL (mmol/L) (mean ± SD)	1.04 ± 0.24	1.64 ± 0.99	−1.461	0.045
PHR (PLT/HDL)	227.60 ± 77.61	207.81 ± 115.70	−2.144	0.033
LDL (mmol/L) (mean ± SD)	3.03 ± 0.88	2.79 ± 1.06	−2.684	0.007
PTA (%) (mean ± SD)	100.13 ± 13.77	99.34 ± 14.76	−0.616	0.538
INR (mean ± SD)	0.99 ± 0.08	1.57 ± 6.48	1.280	0.201
Hepatic stiffness (mean ± SD)	9.13 ± 4.99	9.69 ± 7.62	0.919	0.359
HbA1c (%) (mean ± SD)	5.60 ± 1.16	5.72 ± 1.20	1.071	0.285
GA (mmol/L) (mean ± SD)	13.13 ± 3.00	13.80 ± 3.97	2.017	0.044
Insulin (μU/mL) (mean ± SD)	21.51 ± 9.36	14.54 ± 2.00	−1.004	0.317
C-peptide (ng/mL) (mean ± SD)	4.26 ± 2.19	4.48 ± 3.24	0.797	0.426
Smoking	164 (79.23%)	275 (83.33%)	1.438	0.231
Family history of diabetes	174 (84.06%)	256 (77.56%)	3.350	0.067
Family history of hypertension	160 (77.29%)	269 (81.52%)	10.800	0.001
Good economic status	98 (47.34%)	138 (41.82%)	1.576	0.209
Sedentary lifestyle	8 (3.86%)	19 (5.76%)	0.954	0.329
High caloric diet	30 (14.50%)	94 (28.48%)	14.023	0.000
Taking lipid-lowering drugs	6 (2.90%)	11 (3.33%)	0.078	0.779

ALT, Alanine aminotransferase; AST, aspartate aminotransferase; Tbil, total bilirubin; ALB, albumin; ALP, alkaline phosphatase; GGT, glutamyl transferase; SU, serum urate; GLU, glucose; TC, total cholesterol; TG, triglycerides; HDL, high-density lipoprotein; LDL, low-density lipoprotein; HbA1c, Glycated hemoglobin; GA, Glycated albumin; INR, International normalized ratio; PTA, prothrombin activity; PLT, platelet; PHR, platelet to HDL ratio.

### Correlation analysis of SU levels and MS components in MASLD patients

**Figure 1:**
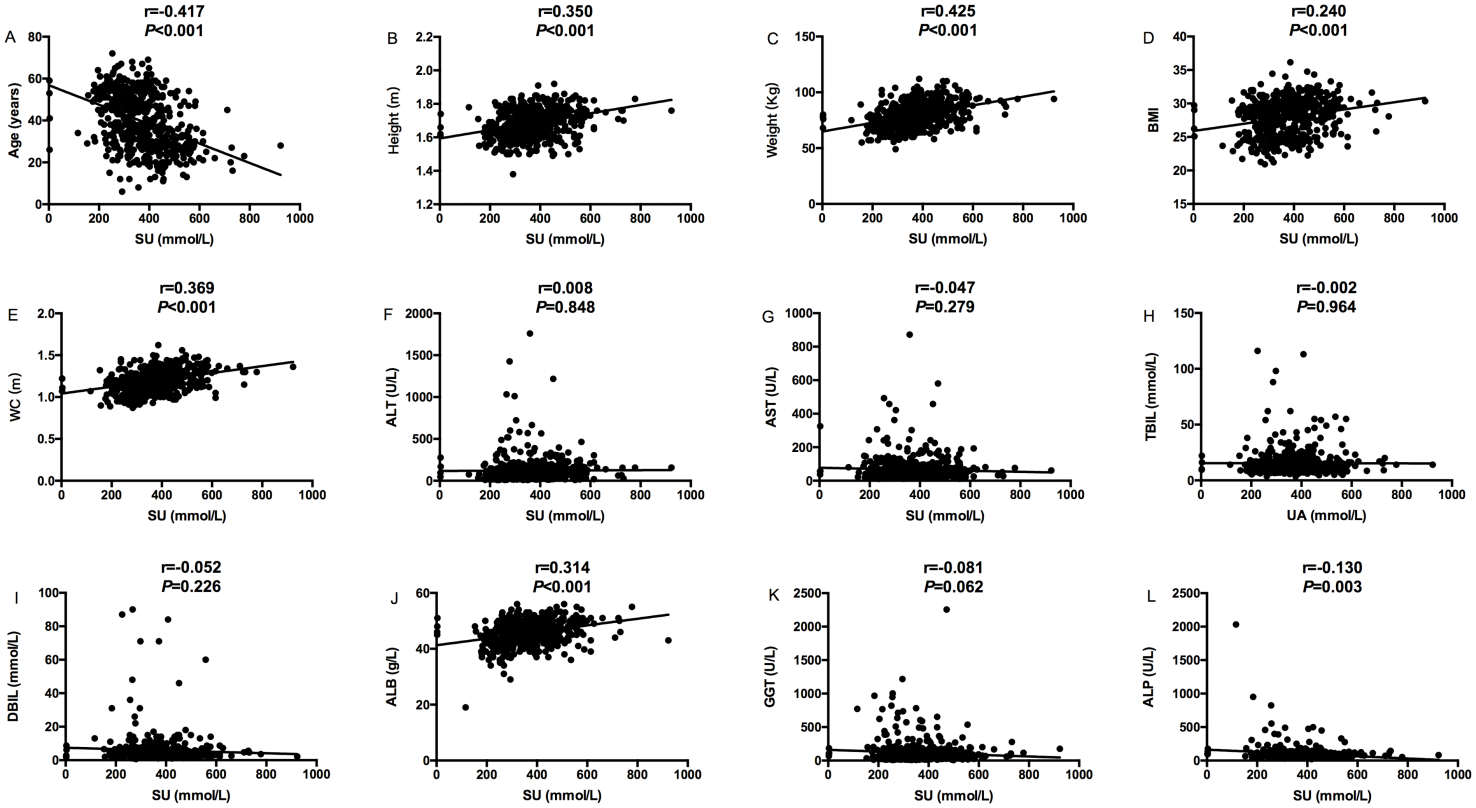
Correlation analysis between serum urate levels and MS components (Age, Height, Weight, BMI, WC, ALT, AST, TBIL, DBIL, ALB, and GGT); **(A)** The correlation between serum urate levels and age; **(B)** The correlation between serum urate levels and height; **(C)** The correlation between serum urate levels and weight; **(D)** The correlation between serum urate levels and BMI; **(E)** The correlation between serum urate levels and WC; **(F)** The correlation between serum urate levels and ALT; **(G)** The correlation between serum urate levels and AST; **(H)** The correlation between serum urate levels and TBIL; **(I)** The correlation between serum urate levels and DBIL; **(J)** The correlation between serum urate levels and ALB; **(K)** The correlation between serum urate levels and GGT; (L) The correlation between serum urate levels and ALP.

**Figure 2:**
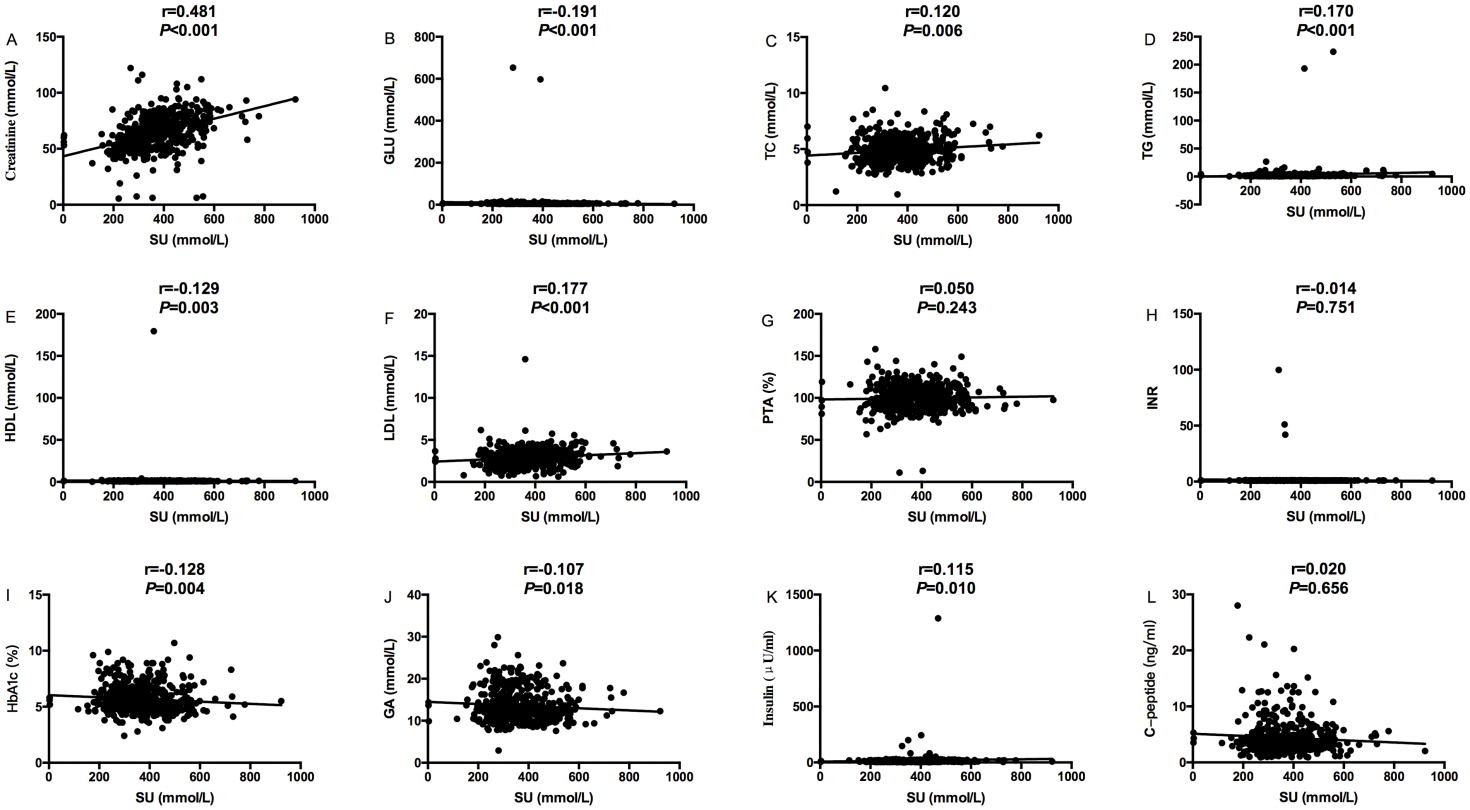
Relevance analysis between serum urate levels and MS components (Creatinine, GLu, blood lipids, PTA, INR, HbA1c, GA, C-peptide and Insulin); **(A)** The correlation between serum urate levels and Creatinine; **(B)** The correlation between serum urate levels and GLU; **(C)** The correlation between serum urate levels and TC; **(D)** The correlation between serum urate levels and TG; **(E)** The correlation between serum urate levels and HDL; **(F)** The correlation between serum urate levels and LDL; **(G)** The correlation between serum urate levels and PTA; **(H)** The correlation between serum urate levels and INR; **(I)** The correlation between serum urate levels and HbA1c; **(J)** The correlation between serum urate levels and GA; **(K)** The correlation between serum urate levels and Insulin; **(L)** The correlation between serum urate levels and C-peptide.

The relevance analysis of serum urate (SU) levels and MS components in MASLD patients found that SU levels were positively correlated with height (*p* < 0.001), weight (*p* < 0.001), BMI (*p* < 0.001) and waist circumference (*p* < 0.001), ALT (*p* = 0.005), and ALB (*p* < 0.001) in Figure 1, while SU levels were negatively correlated with age (*p* < 0.001) in Figure 1. SU levels were negatively correlated with blood glucose (*p* < 0.001), and HDL (*p* = 0.003), HbA1C (*p* = 0.004) and glycated albumin (*p* = 0.018) in Figure 2, while SU levels were positively correlated with LDL (*p* < 0.001), creatinine (*p* < 0.001), TC (*p* = 0.006), TG (*p* < 0.001), and insulin levels (*p* = 0.010) in Figure 2 suggesting higher SU level as a result of insulin resistance/hyperinsulinemia might lead to MASLD in patients with MS.

### Risk factor analysis for MASLD with MS

Univariate and multivariate Logistic regression analysis showed risk factors for MASLD with MS. The results showed that age (*p* = 0.031), triglyceride (*p* = 0.002), LDL (*p* = 0.010), glycated hemoglobin (*p* = 0.026), and family history of hypertension (*p* < 0.001) were independent risk factors for MASLD patients combined with MS in Table 3.

## Discussion

MASLD has become a chronic liver disease affecting the health of people (with a prevalence of about 38%) all over the world, and is considered to be the liver manifestation of MS, which is closely related to obesity and the risk of liver complications related to diabetes, hypertension and non-alcoholic steatohepatitis (40, 41). Understanding the pathogenesis and risk factors of MASLD is very important because the number one cause of mortality of MASLD patients is cardiovascular disease (CVD) rather than liver disease (1). MASLD patients typically have one or more MS components, such as hypertension, dyslipidemia, abnormal blood sugar, or insulin resistance (9). As mentioned above, MASLD is correlated to MS, whereas, diabetes, obesity and dyslipidemia are considered as important risk factors of MASLD (42) that could be linked through changes in metabolism. Although liver biopsy is the gold standard to diagnose MASLD, it is an invasive procedure. However, in previous studies, the diagnostic methods for MASLD were mostly based on the combination of medical history, imaging, and biochemical indicators, etc. This retrospective study is based on data collected from MASLD patients who were properly diagnosed through liver biopsy. MASLD occurs when excess fat is accumulated in hepatocyte in the absence of any significant alcohol consumption due to insufficient mitochondrial capacity for beta oxidation. Currently, there is no proper approved pharmaceutical treatment modality for MASLD except recommendation for altering a patient’s predisposing factors, like low-calorie diet and increased physical activity. Many clinical trials are currently under development to find a new promising pharmacological agents for the treatment of MASLD. In a phase 2 trial, for patients with MASH and moderate or severe fibrosis, treatment with tirzepatide for 52 weeks is more effective in relieving MASH without worsening fibrosis (43). Tao et al. 45 have suggested that FOT1 is a promising new treatment option for all stages and future clinical trials of MAFLD (44). Treatment with denifanstat significantly improves the disease activity, MASH resolution, and fibrosis, which supports the entry of denifanstat into Phase 3 development (45). The anti-diabetic medication like pioglitazone (18, 19), and Glucagon-like peptide-1 (GLP-1) receptor agonists (20, 21) have hinted promising results in patients with MASLD. Modest wine (but not beer or liquor) consumption was also suggested for decreased prevalence of suspected MASLD (46) that could be associated with the protective effect of grape-sourced resveratrol. To confirm the therapeutic efficacy of resveratrol for MASLD large-scale randomized controlled trials is necessary.

MS is a group of diseases with multiple components related to each other. It is characterized by three or more of the following conditions: overweight, high waist circumference or obesity, high triglycerides, low high-density lipoprotein cholesterol, abnormal blood glucose, insulin resistance or diabetes, and elevated blood pressure. Our study showed that in MASLD patients, the proportion of male patients was higher than that of female patients. Riazi et al. (47) and Ballestri et al. (48) suggested that the incidence of MASLD in males was higher than that in females, which was consistent with our finding. Our study also showed that age, BMI, and waist circumference in MASLD patients combined with MS were significantly higher than those in patients with MASLD alone. This may be because overweight or obese patients with MASLD are more likely to accompany with MS (49, 50). MS patients may have a history of dyslipidemia, diabetes and hypertension. Our results showed that the levels of cholesterol, low-density lipoprotein, glycated hemoglobin and glycated albumin (51), and family history of diabetes and hypertension in MASLD patients with MS were significantly higher than those in patients with MASLD alone. So MASLD patients with dyslipidemia, diabetes and hypertension were more likely to have MS. In addition, our study showed that the liver stiffness of MASLD patients combined with MS was higher than that of patients with MASLD alone, which may be related to abnormal metabolic factors such as blood glucose, dyslipidemia, and obesity, et al., accelerating the progression of MASLD to liver fibrosis and cirrhosis. Due to abnormal blood lipids in MS patients, the probability of using lipid-lowering drugs may be higher than that of patients with MASLD alone. Logistic regression analysis in this study showed that age, triglycerides, low-density lipoprotein, glycated hemoglobin, and family history of hypertension are independent risk factors for MASLD patients combined with MS (Table 3).

**Table 3:** Analysis of risk factors for MASLD accompanied by MS.

Item	Univariate logistic regression			Multivariate logistic regression		
	OR	95% IC	*p* value	OR	95% IC	*p* value
Male (*n*, %)	0.635	0.449–0.899	0.010			
Age (yrs)	0.968	0.955–0.981	0.000	0.977	0.956–0.998	0.031
Height (m)	6.425	0.855–48.303	0.071			
Weight (kg)	0.987	0.972–1.003	0.105			
BMI	0.872	0.817–0.931	0.000			
WC (m)	0.137	0.035–0.529	0.004			
ALT (U/L)	1.000	0.999–1.001	0.856			
AST (U/L)	0.997	0.995–1.000	0.039			
TBIL (μmol/L)	1.005	0.989–1.020	0.559			
DBIL (μmol/L)	1.002	0.983–1.022	0.835			
ALB (g/L)	0.994	0.955–1.034	0.751			
GGT (U/L)	1.000	0.999–1.001	0.567			
ALP (U/L)	1.000	0.999–1.002	0.677			
Creatinine (μmol/L)	1.007	0.996–1.018	0.225			
SU (μmol/L)	1.000	0.999–1.002	0.836			
GLU (mmol/L)	0.999	0.994–1.003	0.592			
TC (mmol/L)	0.714	0.602–0.847	0.000			
TG (mmol/L)	0.860	0.768–0.963	0.009	0.782	0.669–0.912	0.002
HDL (mmol/L)	0.982	0.932–1.035	0.506			
LDL (mmol/L)	0.626	0.506–0.773	0.000	0.607	0.414–0.889	0.010
PTA (%)	1.004	0.992–1.016	0.519			
INR	0.979	0.937–1.024	0.358			
liver stiffness	0.962	0.934–0.991	0.010			
HbA1c (%)	0.656	0.556–0.774	0.000	0.748	0.579–0.966	0.026
GA (mmol/L)	0.888	0.843–0.936	0.000			
Insulin (μU/mL)	1.001	0.997–1.005	0.558			
C-peptide (ng/mL)	0.960	0.902–1.023	0.207			
Smoking	0.811	0.523–1.257	0.348			
Family history of diabetes	0.310	0.197–0.88	0.000			
Family history of hypertension	0.332	0.211–0.520	0.000	0.317	0.175–0.575	0.000
Good economic status	0.557	0.394–0.788	0.001			
Sedentary lifestyle	0.65	0.293–1.449	0.293			
High caloric diet	0.706	0.470–1.060	0.093			
Taking lipid-lowering drugs	0.262	0.084–0.814	0.021			

ALT, Alanine aminotransferase; AST, aspartate aminotransferase; Tbil, total bilirubin; ALB, albumin; ALP, alkaline phosphatase; GGT, glutamyl transferase; SU, serum urate; GLU, glucose; TC, total cholesterol; TG, triglycerides; HDL, high-density lipoprotein; LDL, low-density lipoprotein; HbA1c, Glycated hemoglobin; GA, Glycated albumin; INR, International normalized ratio; PTA, prothrombin activity.

Insulin resistance (IR) and fat accumulation in the liver are strongly related (52). Multiple studies have shown that insulin resistance/hyperinsulinemia can lead to hyperglycemia, hypertension, dyslipidemia, hyperuricemia, elevated inflammatory markers, and endothelial dysfunction (53). Progression of insulin resistance can lead to metabolic syndrome (MS), metabolic dysfunction associated steatotic liver disease (MASLD), and type 2 diabetes. In this study, among MASLD patients diagnosed through liver biopsy, the weight, BMI, and waist circumference of hyperuricemia patients were significantly higher than patients with normal SU level, suggesting apparent role of higher SU levels (54, 55). However, the difference in serum C-peptide level between MASLD with hyperuricemia group and MASLD with normal SU group is insignificant (4.26 ± 2.19 ng/mL, 4.48 ± 3.24 ng/mL in Table 2) but much higher than normal level (0.9–1.8 ng/mL) suggesting hyperinsulinemia/insulin resistance but not SU level is likely to be correlated with MASLD. Thus, progression of IR most likely leads to MS and MASLD.

In summary, this retrospective study was intended to find the correlation between SU levels or IR and MASLD in patients (diagnosed with liver biopsy) with MS, as well as the independent risk factors for MASLD in patients with MS. After thorough analyzes, it is concluded that IR/hyperinsulinemia but not SU level has closer correlation with MASLD in patients with MS than patients with MASLD alone. Older age, overweight or obesity, higher HbA1C and glycated albumin levels, higher LDL levels and hyperuricemia caused by IR most likely lead to MASLD in older patients with family history of diabetes and hypertension.

## Data Availability

The original contributions presented in the study are included in the article/supplementary material, further inquiries can be directed to the corresponding authors.
